# Editorial: Prosocial and antisocial behavior and personality

**DOI:** 10.3389/fpsyg.2023.1285957

**Published:** 2023-10-09

**Authors:** Wong Ming Wong

**Affiliations:** International College, Krirk University, Bangkok, Thailand

**Keywords:** mental health and psychological wellbeing, prosocial behavior, dark personality traits, motivational factors, technological mediation

This specialized research section aims to advance the scholarly discourse on personality and social psychology by disseminating rigorous, multidisciplinary investigations. The area is committed to a wide range of subject matter, encompassing everything from foundational psychometric paradigms to complex social interaction dynamics. Studies submitted should adhere to high methodological and statistical rigor standards, and a predilection exists for research employing a multi-methodological approach and diverse participant samples.

Consequently, this review aspires to synthesize insights derived from 13 articles. Each probes various dimensions of human behavior, such as psychological wellbeing, altruistic inclinations, malevolent personality constructs, motivational antecedents, and the technological modulators of behavior.

## Mental health and psychological wellbeing: a social context

This section explores adult friendships and romantic relationships' significant influence on mental health and stress levels (Chen et al.; Li and Chu; Wijaya et al.; Zhang Q. et al.; Zhang Z. et al.). Additionally, the importance of mindfulness and its effectiveness in managing symptoms of depression and anxiety are discussed.

## Understanding prosocial behavior: mechanisms and outcomes

Research indicates the importance of prosocial motivations in workplace innovation, particularly in facilitating basic and applied research behaviors (Li, Mao, et al.; Li, Zhou, et al.; Lu et al.). In addition, how exposure to prosocial media content can influence subsequent prosocial behaviors among adolescents is analyzed, emphasizing the moderating role of empathy and moral elevation.

## The social consequences of dark personality traits

The darker aspects of personality, including traits like Machiavellianism, psychopathy, and sadism, have significant social consequences (He et al.; Pineda et al.; Zheng et al.). Mainly, such characteristics are predictive of being victims in bullying scenarios, especially among adolescents.

## The role of motivation in shaping behavior and orientation

This segment highlights the influence of motivational aspects on academic achievements and how mindfulness upbringing and prosocial motivation impact social entrepreneurship orientation (Mauduy et al.; Shan and Tian).

## The technological mediation of social behavior

The rise of new media platforms increasingly impacts prosocial behavior (Li, Mao, et al.; Li, Zhou, et al.). This section examines how different outcomes for prosocial behavior in short videos can influence subsequent prosocial actions, mainly through mechanisms like moral elevation.

## Conclusion

This academic review offers a comprehensive overview of the psychological and social factors that impact human behavior across various contexts, from personal relationships to the workplace.

## Author contributions

WW: Conceptualization, Writing—original draft, review, and editing.

